# Forehead Shape Analysis following Surgical and Conservative Treatment in Metopic Synostosis: A 3-Dimensional Photogrammetry Analysis

**DOI:** 10.1097/PRS.0000000000011753

**Published:** 2024-09-23

**Authors:** Pauline A. E. Tio, Tareq Abdel Alim, Gennady Roshchupkin, Sarah L. Versnel, Mieke M. Pleumeekers, Marie-Lise C. van Veelen, Irene M. J. Mathijssen

**Affiliations:** Rotterdam, the Netherlands; From the Departments of 1 Plastic and Reconstructive Surgery; 2 Neurosurgery; 3 Radiology and Nuclear Medicine; 4 Epidemiology, Erasmus University Medical Center; 5 Child Brain Center, Erasmus MC Sophia Children’s Hospital.

## Abstract

**Background:**

The aim of this study was to describe and compare head shape in surgically and conservatively treated patients with isolated metopic synostosis using 3-dimensional photogrammetry.

**Methods:**

A retrospective review (2017 through 2020) of consecutive patients, age 4 years, with isolated metopic synostosis based on 3-dimensional photogrammetry was conducted. Images were aligned using a template based on healthy age-matched controls, and mean head shapes were reconstructed to evaluate shape development. A comparative subanalysis based on phenotype was performed between patients who were treated surgically and those who were treated conservatively.

**Results:**

A total of 44 patients with isolated metopic synostosis were included: 22 received conservative treatment and 22 underwent fronto-orbital advancement. At 4 years of age, the surgical group showed retrusion of the complete frontal area, whereas the conservative group showed a slight frontal prominence. Both groups showed temporal depression with respect to the controls. In the subanalysis, a similar degree of temporal depression was observed between surgical and conservative treatment. Head shape patterns showed considerable similarity across all severity phenotypes.

**Conclusions:**

This study shows a deviation in forehead shape from normal controls in patients with metopic synostosis following both surgical and conservative treatment by age 4 years. Comparison between surgical and conservative treatment shows a similar degree of temporal depression, a slight prominence in the center of the forehead in the conservative group, and retrusion of the entire frontal area in the surgical group. This observed difference is of considerable similarity across all severity types.

**CLINICAL QUESTION/LEVEL OF EVIDENCE:**

Therapeutic, III.

Metopic synostosis, which results in a trigonocephalic head shape, denotes premature closure of the metopic suture, and stands as the second most prevalent form of single-suture craniosynostosis, occurring in approximately 1 out of 4500 live births.^1,2^ Clinical characteristics include a triangular shaped forehead, sutural ridge, bitemporal hollowing, hypotelorism, biparietal widening, and elevated lateral canthal angles.^3,4^ A variable spectrum of deformities may result, ranging from a benign metopic ridge to severe cases of trigonocephaly.^5^ Metopic synostosis is associated with an increased risk for cognitive delay, behavioral issues, and ocular anomalies.^6–9^ In contrast with other single-suture craniosynostoses, increased intracranial pressure sporadically occurs in patients with metopic synostosis.^10,11^

Surgical correction has been the standard treatment for metopic craniosynostosis, including 2 primary techniques: fronto-orbital advancement (FOA) before age 1 year and endoscopic strip craniectomy with helmet therapy at age 3 to 5 months. However, because of sporadic occurrence of increased intracranial pressure and prevalent cognitive delays and behavioral concerns after surgical intervention, debate persists regarding the criteria for surgical indication, especially in mild to moderate cases.^12^ Long-term outcomes after surgical intervention often include temporal depressions and forehead irregularities, prompting some caregivers of these patients to opt for secondary recontouring procedures.^11,13,14^ Blum and colleagues^15^ report that in young non-surgically treated patients, as age progresses, the severity of the metopic synostosis on computed tomography (CT) scanning decreases, potentially leading to self-correction of the characteristic forehead shape and improvement of aesthetic features.^16^ The extent of this self-correction remains unknown.

Over the past few years, use of 3-dimensional (3D) photogrammetry has increased, gaining interest because of its reliability in volumetric analysis and offering an accessible and radiation-free alternative for follow-up in patients with craniosynostosis.^17^ Recent studies have used 3D photogrammetry for head shape analysis in patients with metopic synostosis.^18–24^ However, these studies primarily focus on surgically treated patients, lacking analyses of conservatively managed cases or comparison between the 2 groups.

The aim of this study was to analyze and compare the head shape of surgically and conservatively treated patients with isolated metopic synostosis using 3D photogrammetry. To further understand forehead shape development, 3D photogrammetry results of both groups were compared with a normal age-matched template.

## PATIENTS AND METHODS

### Study Design and Patients

A retrospective study was performed on all patients with isolated metopic synostosis born between 2017 and 2020 and treated at Sophia Children’s Hospital, Erasmus Medical Center. Since 2017, parents of patients with metopic synostosis at our center have been well educated on the options and offered craniofacial surgery or conservative treatment. 3D photogrammetry is used for the follow-up of these patients with the 3dMDhead system. Regardless of the initial treatment choice, patients were included if they met the following inclusion criteria: diagnosis of isolated metopic synostosis and more than 3 years of follow-up with 3D photogrammetry. According to our protocol at that time, all surgically treated patients underwent FOA, as previously described.^1^ Patients with 3D images of inadequate quality or artifacts hindering head shape analysis were excluded.

Ethical approval was acquired through the ethics committee of Erasmus Medical Center (MEC-2016-312).

### Severity Assessment

Baseline severity was assessed with 2 different measurements. First, severity was determined on the basis of a 2-dimensional (2D) photograph score for patients with metopic synostosis, previously described by Gaillard.^25^ The overall phenotype was graded on a 4-point ordinal scale: normal, 0; mild, 1; moderate, 2; and severe, 3. Three craniofacial plastic surgeons (M.M.P., M-L.C.v.V., and I.M.J.M.) scored all photographs. The overall phenotype severity was assessed at first presentation and used to divided patients into phenotype severity groups (mild, moderate, and severe). Second, the interfrontal angle (IFA) was measured on all available preoperative scans, previously described by Kellogg et al.^26^ IFA was measured in all patients with available CT scans or blackbone magnetic resonance imaging scans of sufficient quality. Participants were measured twice, with an intraclass correlation coefficient of 0.97, indicating excellent intrarater reliability. The IFA was used to asses objectively whether there were differences between the surgically treated patients and conservatively treated patients within the same phenotype group (mild, moderate, or severe).

### Data Acquisition and Processing

Data were acquired using a 360-degree photogrammetry setup (3dMDhead) with recording capabilities. After acquisition, data were labeled and globally aligned using CraniumPy v0.4.2.^27^ The closest age-matched normal head models from a healthy statistical-shape model were subsequently used as templates in a nonrigid registration step.^28^ This step is crucial in computing averages, because it fixes the topologic structure of each mesh to match that of the age-matched template. A heat map was generated comparing the mean shapes of the surgical and conservative groups. Heat maps per phenotype group (mild, moderate, and severe) were generated and compared for the subanalysis.

### Mean Shape Generation

After nonrigid registration, each index position on the head corresponds to approximately the same anatomic structure. The arithmetic means of the *x*, *y*, and *z* coordinates of all vertices are computed to determine the center of mass for each template $\left(T_{CoM}\right)$. After this, the distance from each point on the template $\left(T_{i}\right)$ to its corresponding point on the patient mesh $\left(P_{i}\right)$ is calculated with respect to $T_{CoM}$. The difference between these distances represents the distance $\left(d_{i}\right)$ between the patient mesh and its age-matched template, calculated as follows:


$$\begin{array}{l} d_{i}=\sqrt{{\left(P_{i}-T_{CoM}\right)}^{2}}-\sqrt{{\left(T_{i}-T_{CoM}\right)}^{2}} \end{array}$$

### Statistical Analysis

Statistical analyses were performed using R version 4.4.0. Descriptive statistics were described as mean with SD for normally distributed continuous data and as median with interquartile range (IQR) for nonnormally distributed data. Differences in severity were compared using the *t* test or the Mann-Whitney *U* test, depending on whether the assumption of normality was violated. Spearman rank correlation was used to determine the correlation between the photograph score severity and IFA.

## RESULTS

### Study Population

One patient was excluded because of artifacts hindering head shape analysis on 3D photogrammetry. Patient characteristics are presented in Table 1. In total, 44 consecutive patients with isolated metopic synostosis were identified. Among these patients, 31 (70.5%) were male. A total of 22 patients received surgical treatment, and 22 patients received conservative treatment. The median age at the time of 3D photogrammetry was 52.5 months (IQR, 49.3 to 56.8) for the surgical group and 51 months (IQR, 48 to 56) for the conservative group. For the surgical group, the mean age at the time of FOA was 9.5 ± 0.9 months.

**Table 1.** Patient Characteristics

Characteristics	Surgical	Conservative	Total
No. of patients (%)	22 (50)	22 (50)	44
Male, no. (%)	18 (81.8)	13 (59.1)	31 (70.5)
Mean age at surgery (±SD), mo	9.5 ± 0.9	—	—
Mean age at follow-up (IQR), mo	52.5 (49.3–56.8)	51 (48–56)	52 (48–56.3)
Severity score at presentation, mean ± SD	2.3 ± 0.8	1.6 ± 0.8	—
Mild	5	12	17
Moderate	5	6	11
Severe	12	4	16

### Severity Assessment

**Fig. 1.**
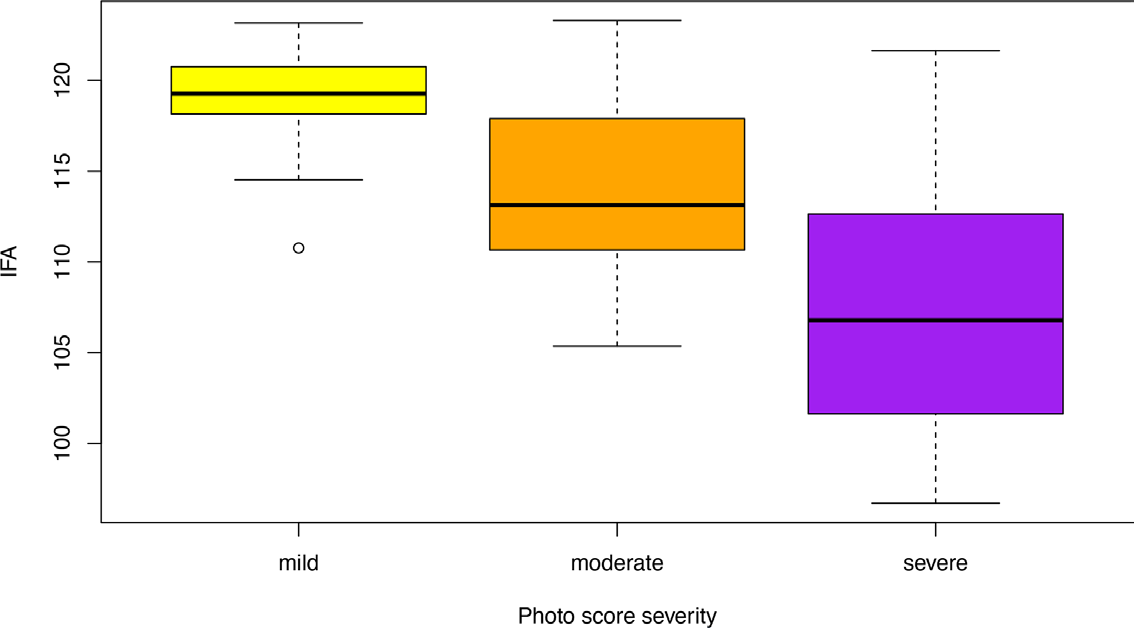
Box plot of the IFA by photograph score severity: mild (*yellow*), moderate (*orange*), or severe (*purple*).

The overall phenotype severity photograph score was determined for all patients at initial presentation. At the time of first presentation, there was a mean (±0.8) overall phenotype score of 2.3 in the surgical group and 1.6 (±0.8) in the conservative group. Statistical analysis (Mann-Whitney *U* test) showed that overall phenotype between the groups at time of presentation was significantly different (*P* = 0.01). The IFA was measured in 38 patients. Radiologic imaging was of poor quality or unavailable in 6 patients (2 surgical and 4 conservative). There was a moderate positive correlation between the photograph severity score and the IFA, which was statistically significant (rho = 0.4, *P* = 0.008) (Fig. 1).

### Forehead Shape: Surgical Group versus Age-Specific Normal

**Fig. 2.**
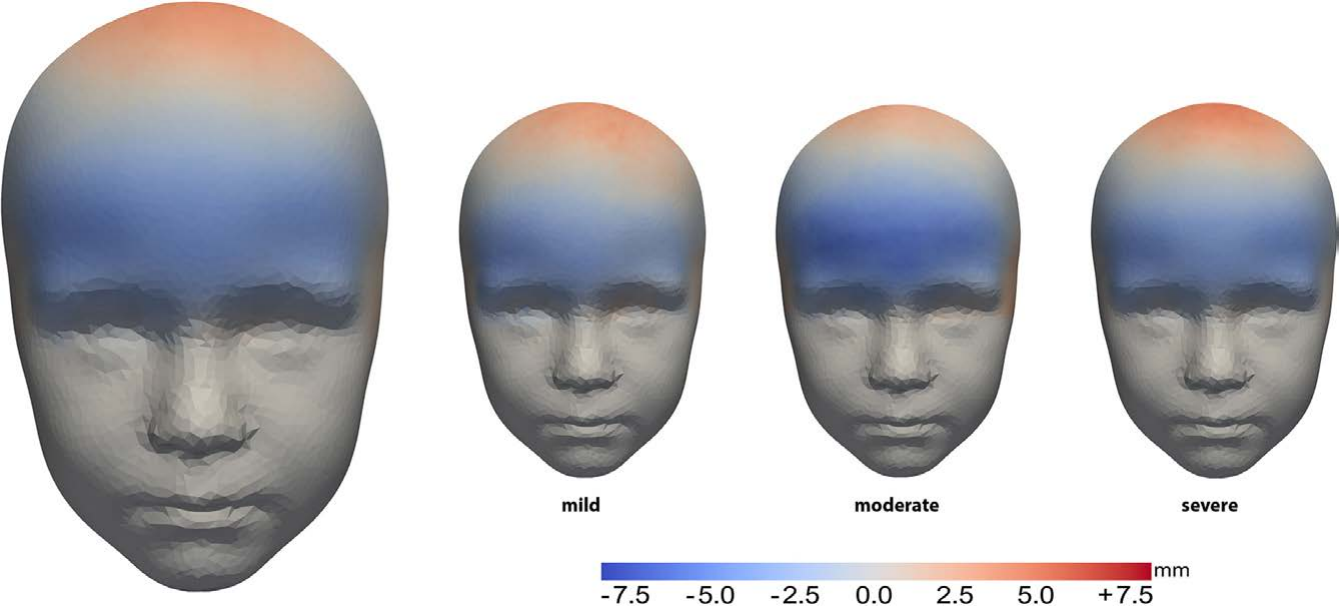
Mean forehead shape in the overall surgical group (*left*) and based on severity (mild, moderate, or severe) (*right*) compared with a normal head model. Heat maps show deviations from the normal model, with *red* areas indicating outward protrusions and *blue* areas indicating indentations.

Figure 2 shows the mean head shape of the surgical group compared with the age-specific normal template. The heat map shows depression of the temporal area after FOA. Retrusion of the complete frontal area was also seen in these patients.

### Forehead Shape: Conservative Group versus Age-Specific Normal

**Fig. 3.**
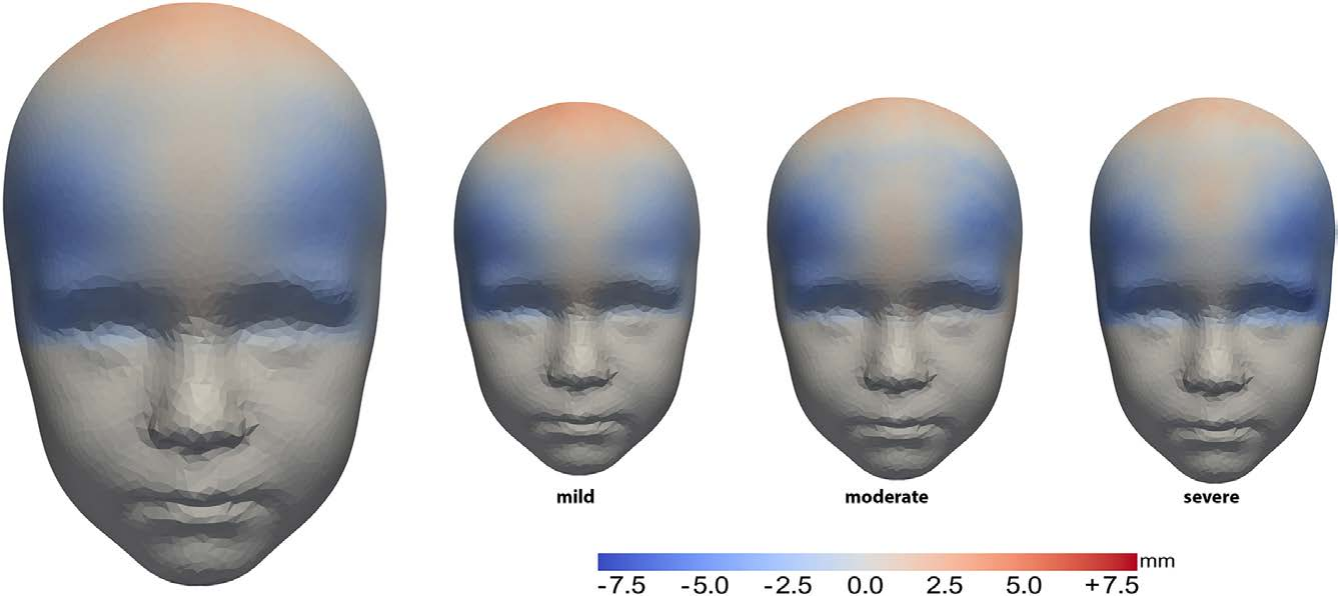
Mean forehead shape in the overall conservative group (*left*) and based on severity (mild, moderate, or severe) (*right*) compared with a normal head model. Heat maps show deviations from the normal model, with *red* areas indicating outward protrusions and *blue* areas indicating indentations.

Figure 3 shows the mean head shape of the conservative group compared with the normal template. The conservative group had a slight wedge in the center of the frontal bone compared with the normal template. Depression of the temporal areas was also seen in these patients.

### Subanalysis: Comparison of Forehead Shape in the Surgical and Conservative Groups

**Fig. 4.**
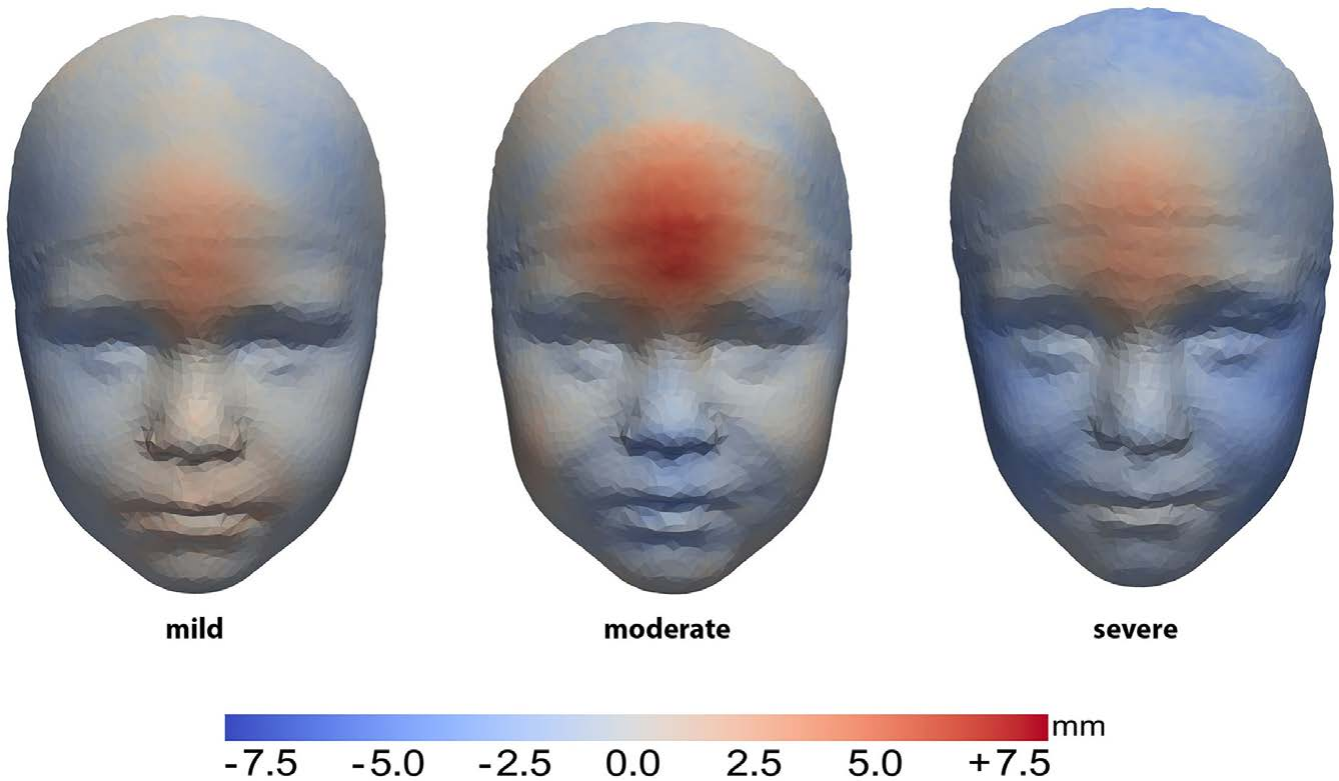
Mean forehead shape in conservatively treated patients compared with surgically treated patients. Heat map shows deviations from the surgical group, with *red* areas indicating outward protrusions and *blue* areas indicating indentations.

To analyze patients with comparable severity at baseline, patients were divided on the basis of the overall phenotype severity score. Based on this classification, the subanalysis for the mild phenotype includes 17 patients (5 surgical and 12 conservative); for the moderate phenotype, 11 patients (5 surgical and 6 conservative); and for the severe phenotype, 16 patients (12 surgical and 4 conservative). There was no significant difference in IFA between patients treated surgically or conservatively in the mild, moderate, or severe group (*P* = 0.214, *P* = 0.052, and *P* = 0.177, respectively) (Table 2). The mean head shape of the conservative group compared with the surgical group per phenotype severity is shown in Figure 4.

**Table 2.** Interfrontal Angle per Severity Group

Severity	Surgical Group	Conservative Group	*P * ^a^
	*Median (IQR*)		
Overall	111 (97–125)	116 (108.99–123.01)	0.196
Mild	116 (110.17–121.83)	120 (118.26–121.74)	0.214
Moderate	119 (112.74–125.26)	112 (108.86–115.14)	0.052
Severe	105 (94.3–115.7)	113 (109.76–116.24)	0.177

a Mann-Whitney *U* test.

A similar degree of temporal depression was observed between the 2 groups. Following conservative treatment, patients had a more anterior placed frontal area compared with the surgically treated patients. Although there is considerable similarity across all severity types, this effect was most prominent in the moderate group.

## DISCUSSION

The primary objective of our study was to compare forehead shape in patients with isolated metopic synostosis treated surgically or conservatively using 3D photogrammetry with age-matched norms and with each other. After FOA, at age 4 years, 3D analysis showed temporal depression and retrusion of the complete frontal area compared with healthy controls. Conservatively treated patients displayed a slight prominence in the midline and temporal depression at age 4 years compared with healthy controls. To compare groups with equal severity at first presentation, a subanalysis was performed dividing patients with metopic synostosis on the basis of baseline severity. There was no significant difference within each severity group in IFA between surgically and conservatively treated patients. Although both groups exhibited similar degrees of temporal depression, the surgical group presented a noteworthy retrusion of the overall frontal area, and the conservative group demonstrated a slight frontal prominence. Among the different severity groups, these observed differences showed similar patterns, but were most evident in the moderate-severity group.

There was a statistically significant difference in severity at presentation between the overall surgical and conservative group based on the photograph score. Since 2017, our center has adopted a shared decision-making protocol for treating metopic synostosis. With this approach, the treatment decision is made by parents in agreement with their clinician. Our hypothesis is that the difference in severity can be explained because parents of children with a more severe presentation favored surgical intervention.

In the literature, the recurrence of temporal depression is described in 18% to 77% of cases based on subjective observations, and its recurrence increases with a longer follow-up time.^11,13–15^ Therefore, a subsequent secondary recontouring procedure is requested in 0% to 5% of cases.^11,13–15,29^ However, this might be an underestimation, as the average follow-up time in most studies ends years before adolescence.^13,15^ In our cohort, both surgically and conservatively treated patients showed temporal depression at age 4 years, with minimal differences between the 2 groups in the degree of depression. While FOA in patients with metopic synostosis aims to address aesthetic concerns, the frequent postoperative recurrence of temporal depression questions its viability as a fundamental surgical practice.^11,13–15^

Notable retrusion of the complete frontal area after FOA was found in our study. In their research using 3D surface scans, Rodriguez-Florez and colleagues^22^ showed that after FOA, at age 17 months, the complete frontal area was retruded compared with controls, despite overcorrection. In addition, they noted that controls had a more rounded forehead, and the postoperative forehead shape in patients with metopic synostosis was flatter. This observed flatness after FOA could describe the difference in forehead shape between the surgical and conservative groups. Pressler and colleagues^18^ reported similar findings, demonstrating minimal frontal anterior growth after open cranial vault remodeling over several time points: 1 month postoperatively, 10 to 14 months postoperatively, and 2 years postoperatively. In contrast, there was evident growth following strip craniectomy over the same periods. The minimal improvement in the axial contour of the forehead following open cranial vault remodeling suggests reduced frontal growth compared with patients who underwent strip craniectomy. The same phenomenon of no difference in forehead contour between the immediate postoperative and 1-year postoperative period following open cranial vault remodeling is described by Linden and colleagues.^20^ Volume loss of the frontal area following FOA compared with controls at age 21 months is described by Schulz and colleagues.^30^

In addition, worsening of anthropometric measurements in patients with metopic synostosis following FOA has been described in the literature. Kuehle and colleagues^31^ measured the frontal angle and interfrontoparietal: interparietal ratio in these patients to describe the narrowing of the frontotemporal region and the rounding of the forehead. Both measurements increased from preoperatively to 14 days postoperatively. Both measurements decreased between 14 days and 12 months postoperatively, with a significant decrease in the interfrontoparietal: interparietal ratio. Although no explanation was provided, this may suggest that recurrence of the initial deformity during the postoperative follow-up can occur promptly.

Although the retrusion of the frontal area after FOA is likely multifactorial, we hypothesize that the effect of FOA on disrupting coronal sutures before age 1 year and bone manipulation interferes with the growth perpendicular to the coronal sutures. This hypothesis is supported by the differences observed between open cranial vault procedures and strip craniectomy, but further investigation is required.

Our findings show the presence of trigonocephalic characteristics in conservatively treated patients by age 4 years. Although there is only a slight frontal ridge compared with healthy controls, the temporal depression is still evident. Because of the cross-sectional data, no conclusions can be drawn on the self-correcting potential in conservatively treated patients with metopic synostosis. However, in our practice, most parents of conservatively treated patients mention a visible decrease in severity.

Despite surgical aims to correct aesthetic outcomes, our findings question the efficacy of FOA in resolving temporal depression over the long term. Similar degrees of temporal depression in the surgical and conservative groups challenges the notion of surgical superiority in addressing the characteristic aesthetic concerns associated with metopic synostosis. Moreover, the observed retrusion of the complete frontal area in the surgical group raises concerns about the overall merit of surgery compared with conservative management. However, by age 4 years there remains a slight forehead prominence after conservative treatment. We hypothesize that because the head growth has not reached its full potential by this age, aesthetic characteristics will further improve over time in conservatively treated patients with metopic synostosis.

Our research is not without limitations. First, although for a rare disease our sample is reasonably large, the sample in our subanalysis is underpowered, which should be addressed in future studies. As a result, patients in the surgical group with moderate severity appear to have a greater degree of frontal retrusion. This difference should be assessed with caution due to the subgroup sample size. With this work we aimed to describe the global patterns in forehead shape of patients with metopic synostosis at age 4 years. Second, not all patients had available 3D photogrammetry at age 4 years, which could result in potential selection bias. To decrease the potential of selection bias, patients were matched on the basis of severity for the subanalysis. Third, the 2D photograph score described by Gaillard^25^ to determine the severity at the time of the first presentation is a subjective score, which could result in variations in severity. Only the overall phenotype score was used, because research indicated it had the highest level of agreement. In addition, the IFA described by Kellogg et al.^26^ was used as an objective anthropometric measure. However, this measurement is 2D-oriented and therefore lacks the description of the overall phenotype of patients. Fearon and colleagues^32^ implied that anthropometric measurements alone are insufficient to describe appearance. Our center sees little added value in calculating anthropometric measurements to determine severity, but to align with the literature, we chose to calculate the IFA. However, used in conjunction, the objective anthropometric measurement and the photograph score mutually reinforce one another, thereby improving the accuracy in assessing severity. Preferably, tools for an objective assessment of severity based on 3D photogrammetry are available. A validated machine-learning algorithm, CRANIORATE, is used for assessing the severity of metopic synostosis through CT scans.^15^ Its adaptation to 3D photogrammetry holds significant promise in enabling a more objective assessment of severity, thereby amplifying its importance in this domain and advocating its practical application in clinical practice.^33^ In addition, it is not uncommon for 3D photogrammetry data to have artifacts, which can affect the visualization of the head shape. Hair is a common cause for artifacts. Nylon caps are used to minimize the artifacts caused by hair. Images with large artifacts were not included in this study. However, an uncertain degree of interference can result from certain hairstyles or a large amount of hair.

Future directions of research should focus on the evaluation of head shape in conservatively treated patients over a longer period of time with sequential data, which is necessary to increase understanding of the self-correcting tendency, and include parental observations. A 3D shape analysis comparing FOA, endoscopic strip craniectomy with helmet therapy, and conservative treatment will follow. In addition, studies focusing on the effect of surgery in adolescence are scarce, but necessary to understand the long-term effect of craniofacial surgery.

## CONCLUSIONS

This study demonstrates that the forehead shape of conservatively as well as surgically treated patients with isolated metopic synostosis deviates from age-specific normal controls at age 4 years. Comparison between surgical and conservative treatment shows a similar degree of temporal depression, a slight prominence in the center of the forehead in the conservative group, and retrusion of the entire frontal area in the surgical group. This observed difference is of considerable similarity across all severity types.

## DISCLOSURE

The authors have no financial relationships or conflicts of interest to disclose.
